# Impact of community-based interventions on maternal and neonatal health indicators: Results from a community randomized trial in rural Balochistan, Pakistan

**DOI:** 10.1186/1742-4755-7-30

**Published:** 2010-11-05

**Authors:** Farid Midhet, Stan Becker

**Affiliations:** 1 Department of Family and Community Medicine, Qassim University College of Medicine, Buraydah, Saudi Arabia; 2 Department of Population and Family Health, Bloomberg School of Public Health, Johns Hopkins University, 615 North Wolfe Street, Baltimore, Maryland, USA 21205

## Abstract

**Background:**

Pakistan has high maternal mortality, particularly in the rural areas. The delay in decision making to seek medical care during obstetric emergencies remains a significant factor in maternal mortality.

**Methods:**

We present results from an experimental study in rural Pakistan. Village clusters were randomly assigned to intervention and control arms (16 clusters each). In the intervention clusters, women were provided information on safe motherhood through pictorial booklets and audiocassettes; traditional birth attendants were trained in clean delivery and recognition of obstetric and newborn complications; and emergency transportation systems were set up. In eight of the 16 intervention clusters, husbands also received specially designed education materials on safe motherhood and family planning. Pre- and post-intervention surveys on selected maternal and neonatal health indicators were conducted in all 32 clusters. A district-wide survey was conducted two years after project completion to measure any residual impact of the interventions.

**Results:**

Pregnant women in intervention clusters received prenatal care and prophylactic iron therapy more frequently than pregnant women in control clusters. Providing safe motherhood education to husbands resulted in further improvement of some indicators. There was a small but significant increase in percent of hospital deliveries but no impact on the use of skilled birth attendants. Perinatal mortality reduced significantly in clusters where only wives received information and education in safe motherhood. The survey to assess residual impact showed similar results.

**Conclusions:**

We conclude that providing safe motherhood education increased the probability of pregnant women having prenatal care and utilization of health services for obstetric complications.

## Background

More than sixty years after independence, Pakistan has shown very little improvement in the health status of its population and is lagging far behind its immediate neighbours (except Afghanistan) in terms of health and social indicators. Of particular concern is the lack of progress in maternal and child health (MCH). Results of the Demographic and Health Survey (DHS) indicate that progress in the MCH indicators over the past decade has remained slow and that Pakistan's prospects for meeting the Millennium Development Goals (MDGs) in MCH are bleak. The maternal mortality ratio (MMR) in Pakistan is estimated at 276 per 100,000 live births [1]. The MMR is significantly higher in the rural areas and in the less developed province of Balochistan.

Maternal mortality remains high in most developing countries though researchers recently have shown a decline from the 1990s [2-4]. The number of maternal deaths worldwide is believed to range between 300,000 and 400,000 annually, 99.8% of them occurring in the developing countries [3]. It is now recognized that progress toward achieving the fifth MDG in the developing countries is slower than anticipated.

It has been argued that lack of involvement of communities in safe motherhood programs is an important reason for persistently high MMR in developing countries. Indeed, a closer look at the 'three delays' model [5] suggests that maternal mortality can be reduced by addressing the delays in decision-making to seek medical care for an obstetric complication and the delays in pre-hospital transportation. The reasons for the first delay (namely: lack of awareness of obstetric danger signs, cultural, social and economic barriers and possibly a lack of trust in the health system) can be addressed through behaviour change communication programs. Similarly, causes of the second delay (lack of access to transport and telecommunication, unfamiliarity with the health system, and financial and social barriers) can be addressed through community mobilization programs. Therefore, both of these delays could be reduced through community-based interventions (CBI) involving women and their husbands, the birth attendants and the community leaders. Regardless of these considerations, country-level evidence accumulated over the last two decades suggests that a reduction in maternal mortality can only be brought about by providing skilled birth attendance and access to emergency obstetric care. What, then, is the role of CBI in this complex problem of multiple dimensions? We address this question in the present paper. We also evaluate the impact of including husbands in the behaviour change communication programs for safe motherhood.

Indirect evidence from descriptive studies shows that husbands play an important role in ensuring better pregnancy outcomes and maternal survival. A study in La Paz, Bolivia, found that husbands' knowledge of obstetric danger signs was associated with high utilization of obstetric care services by women [6]. A study in Maharashtra, India, estimated the risk of maternal death (after adjusting for income and place of residence) to be about three times higher among wives of uneducated men compared to women whose husbands had college education [7].

Husbands are often the decision-makers when it comes to seeking medical care for obstetric complications, or to arrange for transportation. A study in Tanzania [8] found that husbands were responsible for making the decision to seek medical care in 66 out of 117 cases of maternal deaths that were investigated. In a study in Afghanistan, 87% of women had to obtain their husbands' permission before seeking medical care during pregnancy and delivery [9].

Husbands can help reduce maternal mortality and morbidity by: 1) Encouraging and facilitating their wives' use of prenatal care; 2) Ensuring better nutrition and rest for their wives during pregnancy and the postpartum period; 3) Arranging for a skilled birth attendant for delivering the baby; 4) Preparing for the possibility of obstetric emergencies by arranging transportation and finances; and 5) Reducing the delay in the decision to seek medical care in case of obstetric emergencies.

In this paper, we present results from a community-based operations research project to reduce maternal and neonatal mortality in a remote rural district of Balochistan province of Pakistan. The project was designed to develop and implement a CBI package comprising information and education for empowerment and change (IEEC) for women and their husbands, training of birth attendants in early recognition of obstetric danger signs and providing telecommunication and transportation services for women in need of emergency obstetric and neonatal care (EmONC). IEEC is a strategy for approaching women and their husbands with information about safe motherhood. The CBI package addressed the first two delays. In this paper, we test the hypothesis that the CBI package had a significant impact on maternal and neonatal health (MNH) indicators. A secondary hypothesis is that including husbands in the IEEC strategy significantly increased the impact of CBI on MNH indicators.

## Methods

**Figure 1:**
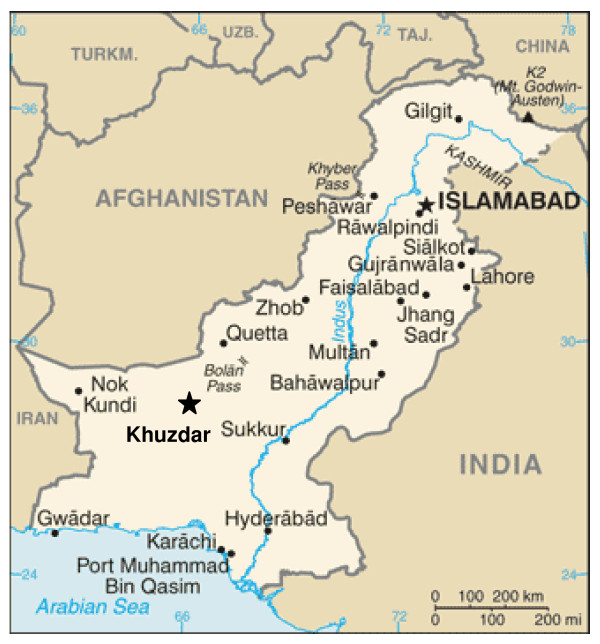
Location map of project area

The project was implemented during 1998-2002 in 32 village clusters in Khuzdar, a rural district of Balochistan province in Pakistan (Figure 1). Balochistan is the most underdeveloped province of Pakistan; approximately 76 percent of the population is in the rural areas. Due to a scattered rural population and absence of proper roads, people's access to health services, particularly hospitals, is extremely limited. In the primitive tribal society, a vast majority of women are uneducated and their mobility is limited. The maternal and child health indicators in Balochistan are the poorest in the country. The maternal mortality ratio (MMR) is the highest in Balochistan (786 maternal deaths per 100,000 live births, compared to the national average of 276 [Pakistan DHS 2007]).

**Figure 2:**
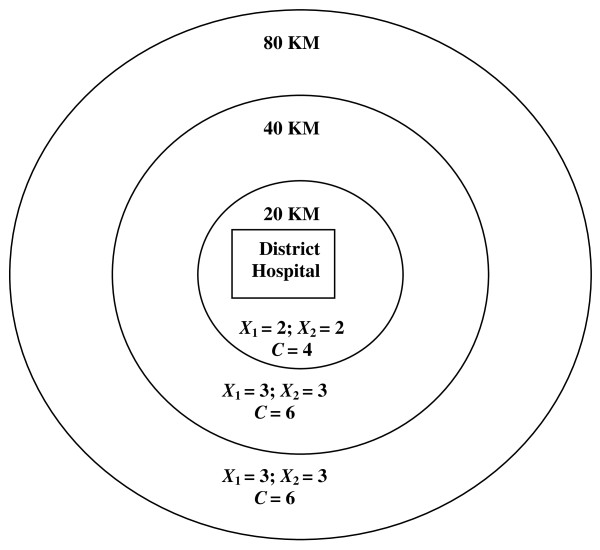
Distribution of intervention and control clusters

**Figure 3:**
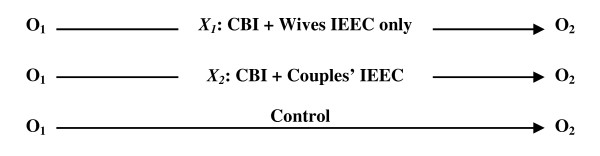
Study design

Each village cluster had 5-15 villages and an average population of 2,000. All village clusters were within 80 kilometres of the central town where the district hospital is located. The project area was divided into three zones: village clusters lying within 20 kilometres of the district hospital (zone 1); those lying between 20 and 40 kilometres (zone 2); and those located between 40 and 80 kilometres away (zone 3) (Figure 2). Each village cluster in zones 2 and 3 was served by a government primary health facility and all villages in the cluster were located within seven kilometres of that health facility. Randomization took place separately within each of the three zones; equal numbers of village clusters were randomly allocated to the intervention or control sites (by blindly drawing village cluster names written on folded chits). Sixteen of the 32 village clusters were thus assigned to the intervention arm while the remaining clusters served as the control arm. In addition, in a randomly selected half (eight) of the intervention clusters, husbands also received IEEC materials that were specially designed for them (Table 1). The impact of interventions on safe motherhood indicators was assessed through pre-intervention (baseline) and post-intervention (follow-up) surveys (Figure 3).

**Table 1:** Description of Interventions

Intervention strategy	Control arm	Intervention arm	
		
		Women's IEEC only	Couples' IEEC
Number of village clusters	16	8	8

IEEC to husbands			✓
IEEC to women		✓	✓
Training of traditional birth attendants		✓	✓
Setting up of transport and telecom systems		✓	✓

### Interventions

The IEEC for women was designed to increase awareness of safe motherhood and neonatal health. The IEEC materials (pictorial booklets and audiocassettes) were developed after formative research and were field tested for suitability to local culture. The project staff then identified ten female volunteers from each village cluster and trained them as IEEC facilitators. Each facilitator initially invited 10-12 women from close villages to participate in a support group. A typical support group started with a discussion of the problems faced by women during pregnancy and childbirth. Participants were then asked to look at their booklets while listening to a cassette tape that guided them through the pictures in the booklet. The pictures formed part of the dramatized stories recorded on the tape, thus creating an audio-visual effect. The booklet covered the following topics: family planning; nutrition; preparation for pregnancy and delivery; and danger signs during pregnancy, delivery and postpartum. Typically, the booklet was finished in six sessions of 1-2 hours each, after which the participants were entitled to have their personal copy of booklet and audiocassette. Outstanding participants were encouraged to organize and facilitate support groups in their own villages; these women formed the 'second-generation' of IEEC Facilitators - as they were not directly trained by project staff. A 'third generation' of IEEC Facilitators also emerged, thus enabling the project to reach about 4,000 women in three months. (Booklets and audio-cassettes are available from the first author).

Local traditional birth attendants (TBAs) - who deliver over 90% of all births in the project area - were trained in clean home delivery and in recognizing common obstetric and newborn emergencies. The project also facilitated timely referral and transportation of obstetric and newborn emergencies to the district hospital. Firstly, local owners/operators of public transport vehicles (pickup trucks) were trained in the use of stretchers and were given an orientation to the district hospital, introducing them to the doctors and other medical staff. Secondly, very high frequency (VHF) wireless telecommunication systems were set up in the village clusters located in zones 2 and 3. These systems linked the TBAs and drivers to the local health facility, the hospital and the project office in Khuzdar. Using this system, the TBAs were able to contact the local health facility and the drivers and could ask for an ambulance or other transportation. (Cell phone services were not available in the project area.)

The husbands' IEEC was implemented in eight village clusters randomly selected from the 16 intervention clusters. Husbands' booklets and audiocassettes were designed after formative research with married men. Then in each village cluster, 20-30 male community volunteers were identified who distributed the materials among husbands of the women who had participated in the support groups.

The district hospital served the entire project area and was staffed and equipped to provide comprehensive emergency obstetric and newborn care (EmONC). The project provided advanced training for obstetricians, paediatricians and anaesthetists working at the district hospital. In addition, government healthcare providers serving in primary health facilities in both intervention and control arms were provided training in clinical skills required for obstetric and neonatal care.

The project was officially launched on January 1, 1998. Formative research and establishing formal relationship with the communities and the government health services took most of the first year. The baseline survey was conducted during August-September 1998. During 1999, the IEEC materials were developed and field-tested. The transport and telecommunication systems were set up in early 2000. By March 2000 the complete CBI package was in place. The follow-up survey was conducted in March-April 2002; the time interval between baseline and follow-up surveys was about 40 months. Between April and September 2002, an expansion of IEEC and TBA training was carried out by a local NGO, covering an additional population of approximately 90,000 in the district. During July-September 2004, (two years after closure of project), the NGO conducted a district-wide sample survey to assess the residual impact of IEEC on selected safe motherhood indicators.

#### Survey Methodology

Baseline and follow-up surveys were conducted by local women having secondary school education or above, who were given an intensive, three-week training. All interviewers spoke the local language. In each household all ever-married women under 50 years of age were interviewed. The survey design was similar for the baseline and follow-up surveys. The questionnaire covered the following areas: 1) A list of household members with age, sex, education and occupation of each member; type of house construction, electricity, telephone connection and tap water; and ownership of valuable items (radio, TV, etc.), farm animals and arable land. 2) For each ever married woman in the household, current age and age at marriage and years of schooling were recorded. A truncated pregnancy history (the last five years) was also recorded. 3) For the women who had a pregnancy during the past 12 months (regardless of the outcome of pregnancy), the following information was recorded: adequacy of prenatal care (visiting a qualified healthcare provider during first or second trimester of pregnancy for routine prenatal check-up) and tetanus immunization history; outcome of pregnancy; diet during pregnancy; details of any illnesses during pregnancy, delivery or the postpartum period; type of birth attendant; and, in case of an obstetric complication, was the woman referred to the district hospital.

#### Sample Size and Sampling

The project was designed as an operations research study to include 32 village clusters, each of which was large enough to provide information on about 100 pregnancies in the year preceding the survey. The initial survey data were used to examine if there were significant differences between the intervention and control arms in terms of socioeconomic profile and maternal and neonatal health indicators. The follow-up survey interviews were completed in 95.2% of the visited households. The main reasons for incomplete interviews were refusal or absence of an eligible respondent.

The district-wide sample survey, conducted by a local NGO during July-September 2004, included 900 households randomly selected from the immunization (EPI) records that were available at the district's health office. The questionnaire elicited the following information on pregnancies ending during the preceding 12 months: use of iron-folic acid during pregnancy; routine prenatal check up during first and/or second trimester of pregnancy; use of skilled birth attendant or trained TBA; did the husband accompany his wife to the health facility for prenatal care and/or for illness during pregnancy; pregnancy outcome and neonatal death. In this survey, refusals or locked households in a selected cluster were replaced by the nearest available household.

Most results presented in this paper are from the follow-up survey while selected results of the post-project survey of 2004 are also included.

#### Data Analysis

All questionnaire responses were entered into database files using Epi-Info. Primary data analysis was carried out using SPSS version 11.5 (2006) [10], while we used Stata version 9 (2005) [11] for advanced analysis. We present cross-tabulations of selected indicators by intervention arms and use Pearson's chi-squared statistic to identify significant differences. Statistical comparisons are made between three arms: 1) the control arm; 2) the women's IEEC only intervention arm and 3) the husbands' IEEC intervention arm. The indicators of impact were: perinatal or neonatal death; use of iron-folic acid during pregnancy; prenatal care; tetanus immunization; and delivery in the district hospital. All of these were binary (yes/no) variables. To adjust for the effects of clustering in the logistic regressions we used the SVY procedure of Stata version 9 (2005) [12]. The only independent variable in the logistic regression models was the intervention status (control, women's IEEC only and wives' and husbands' IEEC). We did not include other covariates because the three arms were quite similar in terms of background variables (see below).

## Results

### Comparison of village clusters at baseline

At the baseline survey, socioeconomic and demographic characteristics of married women in the three arms were similar (e.g., age, age at marriage, parity, number of living children and education levels of the woman and her husband) (Additional file 1). The three arms were also similar in socioeconomic variables at the follow-up survey, except that a higher proportion of households had electricity and telephone in the women's only IEEC intervention arm (not shown).

Numbers of households in the intervention and control arms were approximately equal. During the course of the project, village clusters in the intervention arms were expanded by addition of adjacent villages because the second and third generations of IEEC facilitators (see above) could not be constrained within the seven kilometre boundary of the cluster defined at the baseline survey. As a result, in the follow-up survey, the number of women in the intervention arms was 47% greater than that in the control arm. However, no village in the control clusters was included in the IEEC intervention.

### Comparison of village clusters at follow-up survey

During the year preceding the follow-up survey, 2,561 women became pregnant (Table 2). Fewer women in the women's only IEEC arm reported an illness during pregnancy, childbirth or the postpartum period. The proportion of pregnant women who visited a health facility for any reason was significantly higher in the control arm and the husbands' IEEC arm, compared to the women's only IEEC only arm. Significantly more pregnant women in the intervention arms had tetanus immunization and prophylactic iron therapy. A significantly greater percent of women in the intervention arms reported improved diet in pregnancy and reduced household chores, compared to the control arm (P < 0.05). The percent of pregnant women taking iron and improving their diet was slightly higher in the husbands' IEEC arm, although not significantly so. However, significantly more women in the husbands' IEEC arm reported that they reduced their household chores during pregnancy (P < 0.01). Though a greater percent of pregnant women in the control arm visited a health facility, more women in the intervention arms utilized the district hospital for problems arising in pregnancy, delivery and immediately after delivery. Pregnant women in the husbands' IEEC arm visited the district hospital more frequently than their sisters in the other arms (P < 0.01). Finally, the percent of women delivering in the district hospital was higher in the intervention arms (P < 0.05) (Table 2).

**Table 2:** Percentage of women reporting a pregnancy during the past year who utilized selected health services by study arm, Follow-up Survey 2002

Indicator	Control arm	Intervention arms	
		
		Women's IEEC only	Couples' IEEC
Number of women who became pregnant in the past year	1,022	836	703

**Women who reported an illness during:**			
Pregnancy*	92.1	87.6	90.7
Delivery*	60.8	52.6	60.7
Immediately after delivery (ns)	45.1	40.2	44.3
Postpartum period*	69.2	58.8	62.8
Percent of pregnant women who visited a healthcare provider	60.9	49.1	57.7
**Women who:**			
Received tetanus immunization*	10.6	18.5	12.3
Took iron-folic acid tablets*	22.1	25.0	27.6
Improved diet in pregnancy*	18.0	21.5	23.8
Reduced work in pregnancy*	17.5	18.5	25.3
**Women who visited the District Hospital for treatment of problems during:**			
Pregnancy*	13.3	16.2	22.4
Delivery (ns)	8.7	11.0	12.6
Immediately after delivery*	7.6	13.1	13.8
Postpartum period (ns)	5.1	10.4	8.9
**Place of delivery (Percent):**			
District Hospital*	2.9	3.9	4.1
Any other health facility (ns)	0.9	1.6	1.9

*Differences across sites are statistically significant (P < 0.05, using Pearson's chi-square statistic).

Significantly more pregnant women in the intervention arms received adequate prenatal care (as defined earlier) and tetanus immunization (P < 0.05). The differences between intervention arms were not significant (Table 3).

**Table 3:** Percentage of pregnant women who visited a healthcare provider during pregnancy by type of healthcare received, by study arm, Follow-up Survey 2002

Indicator	Control arm	Intervention arms	
		
		Women's IEEC only	Couples' IEEC
Number of pregnant women who visited a healthcare provider	622	410	406

Reason for visit*:			
Prenatal care only	18.7	30.4	26.5
Illness related with pregnancy	50.2	36.7	43.1
Illness not related with pregnancy	13.7	8.6	12.0
To get TT shot or iron pills	13.3	20.0	15.1
Received adequate prenatal care^$^*	17.9	26.2	25.8

*Differences across sites are statistically significant (P < 0.05).

$Adequate prenatal care is defined as a visit to a qualified healthcare provider solely for the purpose of routine medical check up during first or second trimester of pregnancy.

Perinatal and early neonatal morality rates during the year preceding the follow-up survey were significantly lower in the intervention arms. The early neonatal mortality rate was the lowest in the husbands' IEEC intervention arm (P < 0.05) (Table 4).

**Table 4:** Perinatal, early neonatal and neonatal mortality rates (per 1000 live births), by study arm Follow-up Survey 2002.

Indicator	Control arm	Intervention arms	
		
		Women's IEEC only	Couples' IEEC
Number of live births	895	740	622

Perinatal mortality*	95.6	48.7	67.2
Early neonatal mortality*	39.1	24.3	17.7
Neonatal mortality (ns)	48.0	32.4	30.5

*Differences across sites are statistically significant (P < 0.05).

Results of the logistic regression analysis are presented in Table 5, which gives the adjusted odds ratios for the impact of intervention group on selected outcome variables. Pregnant women in the women's only IEEC intervention arm had double the odds of receiving prenatal care and tetanus immunization and 50% lower odds of having a perinatal death, when compared to women in the control arm (P < 0.05). Pregnant women in the husbands' IEEC intervention arm had twice the odds of receiving prenatal care, compared to women in the control arm (P < 0.05). For all other outcome variables, there are no significant differences between the intervention and control arms or between the two intervention arms.

**Table 5:** Adjusted odds ratios (AOR) and 95% confidence limits indicating impact of intervention on selected safe motherhood indicators, Follow-up Survey 2002

Indicator	Control arm (Reference)	Intervention arms	
		
		Women's IEEC only	Couples' IEEC
Number of women who became pregnant during last one year	1,022	836	703

AOR for pregnant women during the last one year, who:			
Received routine prenatal care	1.00	2.4 (1.4, 4.3)	2.9 (1.6, 5.0)
Received tetanus immunization	1.00	1.8 (1.2, 2.9)	0.7 (0.4, 1.1)
Took iron-folic acid tablets	1.00	1.1 (0.9, 1.5)	1.2 (0.8, 1.8)
Improved diet in pregnancy	1.00	1.2 (0.9, 1.5)	1.2 (1.0, 1.6)
AOR for pregnancy resulting in:			
Perinatal death	1.00	0.5 (0.3, 0.7)	1.4 (0.9, 2.2)
Death within 1^st ^week of life	1.00	0.6 (0.4, 1.0)	0.7 (0.3, 1.7)
Death within 1^st ^month of life	1.00	0.7 (0.4, 1.0)	0.9 (0.5, 1.6)
			
AOR for delivery in District Hospital	1.00	1.3 (0.7, 2.5)	1.3 (0.6, 2.7)
AOR for currently using a modern contraceptive method among married women of all ages	1.00	1.6 (1.0, 2.7)	0.7 (0.5, 1.0)

* Odds ratios are derived from logistic regression analysis using Stata (2006) and are adjusted only for clustering effects.

Two years after the project, women in the IEEC clusters fared better than those in the non IEEC clusters in terms of iron use in pregnancy, antenatal visit during the first/second trimester and the percentage of wives accompanied by husbands to the health facility. Differences for these three indicators are statistically significant (Table 6).

**Table 6:** Indicators from the District-wide Post-Project Survey, July-September 2004, by study area

Indicator	Villages that received no IEEC	Villages that received IEEC	
		
		During intervention phase	After intervention phase
Number of women interviewed	469	136	280
Neonatal mortality (per 1000 live births)	61.0	53.0	51.0
Percent of pregnant women who:	18.3	29.9	38.4
Used iron during pregnancy *			
Had Prenatal check up in 1^st ^/2^nd ^trimester *	12.4	31.6	38.2
Had Birth attended by skilled personnel or trained TBA	11.5	19.9	22.8
Were accompanied by husband to health facility for prenatal check up or treatment *	24.1	27.2	39.0

*Differences across sites are statistically significant (P < 0.05).

### Costs and benefits of the interventions

Although we did not collect data for a cost-benefit analysis of the intervention, a rough estimate of the costs is available: for the IEEC intervention, the total cost of booklets, cassettes, group sessions and training of facilitators, etc., was roughly Rs. 530 (US$ 12) per woman in the target population. The average cost of all other training programs including healthcare providers, *Dais *(traditional birth attendants) and drivers, was Rs. 2,900 (US$ 60) per trainee. The telecommunications systems cost approximately US$ 30,000, which included four base stations with transmission towers and about 100 walkie-talkie instruments provided to drivers and *Dais*.

## Discussion

Though this was a community randomized intervention study, it nevertheless has several limitations. It was difficult to control extraneous factors that may have a direct or indirect impact of the indicators of interest. For example, women in the intervention arms sometimes shared their IEEC materials with the women in the control arm. There were also some reports that several of the TBAs trained by the project were called to deliver babies in control villages. Also in the women's only intervention arm, husbands could always look at their wives' booklets, which might have diluted the effect of explicitly adding the husbands' IEEC in the second intervention arm. However, such 'contamination' would serve to decrease the observed difference between the study arms.

In spite of these limitations, we conclude that community-based interventions do have an impact on neonatal mortality in remote rural areas of Pakistan. First, the women in the women's only IEEC intervention arm reported fewer problems and visited a health facility less frequently than their sisters in the control arm, probably because they were relatively better equipped to identify a serious problem. In the husbands' IEEC arm, on the other hand, more women visited a health facility -- perhaps encouraged by their husbands to see a healthcare provider even for minor problems. Also a significantly greater percent of women in the two intervention arms visited the district hospital for problems related with pregnancy and immediately after delivery. This would have been unlikely without a better understanding of the problem and support from the husband. Second, while the overall percentage of hospital deliveries was low, significantly more women in the intervention arms delivered in the district hospital. The use of prenatal care was also significantly higher in the intervention arms. An added impact of the husbands' IEEC on prenatal care was not observed. Nonetheless, husbands' IEEC resulted in significant improvements in diet and reduced workload of their pregnant wives, more regular use of prophylactic iron and folic acid and more frequent visits to the hospital during pregnancy and delivery.

Historically, studies on maternal mortality have divided the causes of maternal deaths into patient-oriented and hospital-oriented categories. The distinction between deaths among 'booked' and 'un-booked' cases was sometimes used to suggest that pregnant women who did not register for prenatal care were more likely to have complications and die. It was only after the launch of the global safe motherhood initiative [13] that the complex nature of the multiple causes of maternal mortality was recognized. For example, the 'three delays' model [3] identifies the first delay in decision making to seek medical care for obstetric emergencies, which is attributed to lack of awareness. However, another cause of the first delay could be a lack of trust in the health system. Consequently, the strategies to reduce maternal mortality can be classified into community-oriented and health system-oriented categories. This, in part, is an application of the demand and supply theory in maternal health. There is an ongoing debate on which strategies work best to improve the demand and supply of maternal health services. Unfortunately, experimentation with different strategies has been disorganized and lacking an evidence base. Many projects end with only vague conclusions such as maternal mortality is a complex public health problem requiring broad-based improvements in health and social systems [14]. On the other hand, there is little evidence for or against particular sets of interventions to reduce maternal and perinatal mortality at the community or at the health system level [15].

Bullough et al. [16] reviewed the literature on strategies to reduce maternal mortality and found that current strategies were based upon insufficient evidence. In particular, interventions of community mobilization and training of traditional birth attendants were not based upon sound evidence. They argue that safe motherhood interventions are complex public health approaches and quite different from single clinical interventions. The conclusion is that there is insufficient evidence to recommend universally effective interventions to reduce maternal mortality. While a consistent and sustained improvement in the quality of health services will almost certainly reduce maternal mortality, short-term interventions and vertical programs are less likely to achieve the millennium development goals in maternal health. Unfortunately, the maternal health agenda in many developing countries is driven by donor priorities and perspectives. Donors support vertical programs because their monitoring and evaluation is easier and their results are available quickly. But vertical programs may erode the general standards of health systems by diverting resources from overall quality and focusing on a single problem [17].

Delays in accessing medical care during emergencies, which are responsible for the bulk of maternal and neonatal deaths, can be addressed in a number of ways. Increasing awareness of pregnancy danger signs and birth preparedness and streamlining existing transportation systems are the most effective means to address the first two delays. However, their impact is conditional upon availability of good quality health services.

Birth preparedness programs are a way to increase awareness about the dangers associated with a supposedly normal pregnancy and delivery. During 2003-2004, a field trial of the Birth Readiness Package was conducted in the Siraha district of Nepal [18]. This was primarily a program to increase demand, which resulted in a significant increase in the knowledge, and some improvement in maternal practices in the intervention site. However, use of skilled birth attendants and emergency obstetric care remained unchanged, indicating that the problem is not resolved merely by improving the demand for better health services. In fact, while demand is partly associated with supply (i.e. trust in the quality of health services leads to greater demand) it is essential to work on both. The importance of having a two-pronged approach for reducing maternal and neonatal mortality is emphasized by many studies. Darmstadt et al. [19] reviewed the impact of community-based interventions on neonatal mortality in a number of countries. They suggest that in settings with high neonatal mortality, community and family interventions can bring down that mortality effectively, but that a complementary health facility-based care model is also necessary. In Nepal, an educational program in neonatal care for the healthcare providers improved their practices in hospital settings, which led to increased utilization of neonatal services and better chances of survival of the neonates brought to the hospitals [20].

During 2003-2006, the Population Council conducted an operations research study similar to ours in two rural districts of Punjab province of Pakistan [21]. The primary objective of that study was to disaggregate the impact of CBI from the impact of interventions directed at improving the quality of health services. The study had two intervention arms. In one arm, a CBI package similar to that of our study was implemented along with interventions to improve the quality of emergency obstetric care. In the other arm, interventions were directed to improve the quality of emergency obstetric care but no CBI was implemented. Their results indicate a significant reduction in perinatal mortality, attributed to community-based interventions (IEEC to women and husbands, training of skilled birth attendants and setting up transportation systems). Improvements were also found in other process indicators. The project report concludes that: "This intervention package led to declines in maternal and neonatal mortality ... by increasing the proportion of mothers and neonates with serious emergencies who seek appropriate and timely help, and for neonates by improving home care at and after birth. While there is evidence that perinatal mortality declined in the CBI communities, the changes in knowledge and practice that would be expected to lead to this result are not convincing. There are several possible explanations, for example: The measurements of knowledge and practice were not sufficiently accurate or precise to pick up real and important changes where they occurred" [[21]: p. 42, Report 1]. On the other hand, the study did not find an impact of health services interventions (such as training of doctors) on perinatal and neonatal mortality [21].

The primary purpose of our study was to test whether CBI to reduce the delays in seeking medical care for obstetric complications could significantly reduce perinatal and neonatal mortality. The project focused on the first two delays, although attempts were also made to strengthen and improve the EmONC services available at the district hospital, which served the entire project area. Our study provides evidence that the CBI package, along with the interventions addressed at the health facilities, led to a significant reduction in perinatal mortality. The study population was too small to detect significant differences in MMR; however, a number of process and output indicators were significantly improved.

While the role of husbands in family planning has been extensively studied, literature on their role in reducing maternal or neonatal mortality is scanty. There are very few randomized trials or quasi-experimental studies on the impact of involving husbands in IEEC interventions to encourage safe motherhood behaviours. An exception is a study in Mumbai, India, where one out of three pregnant women attending a maternity care clinic was encouraged to bring her husband and then both received education about pregnancy and child-rearing; in a control group, only women received such education [22]. In that study, the intervention group had a significantly lower perinatal mortality rate -- 15 deaths per 1,000 total births versus 35 per 1,000 in the control group (P < 0.001).

In our study, lower levels of perinatal mortality in the intervention sites at the follow-up survey could plausibly be attributed to the interventions. A number of other indicators, including referral to district hospital, use of prophylactic iron and folic acid therapy during pregnancy and prenatal care, were also improved in the intervention sites although the effects became non significant after adjusting for clustering. Although some of these indicators were better in the husbands' IEEC intervention arm, most of the differences were not statistically significant. This could be explained by the fact that even in the women's only IEEC intervention arm, husbands could look into the IEEC materials provided to their wives. Therefore, the statistical effect estimated for the exposure to male IEEC materials might have been diluted.

Finally, the residual impact of the interventions was tested through a post-project survey, almost two years after closure of project. This survey provided evidence that the IEEC impact on perinatal and early neonatal mortality persisted in the village clusters where the CBI was implemented (information to women and husbands, training of traditional birth attendants in clean delivery and recognition of obstetric and newborn danger signs, and setting up transport and telecom systems). Moreover, the IEEC intervention carried out in other village clusters in Khudzar by the local NGO also resulted in a significant reduction in neonatal mortality and a significant increase in prenatal care and iron use during pregnancy.

## Conclusions

The CBI package had an impact on utilization of health services for prenatal care and during obstetric complications. A significant reduction in the perinatal mortality rate was also recorded in the sites where only wives received the IEEC. Therefore, we recommend that all safe motherhood programs should include an IEEC strategy for women.

Although the CBI package included training of traditional birth attendants (Dais), it is not possible to segregate the impact of the training from that of the IEEC strategy. Nonetheless, we recommend that, in areas where there are no skilled birth attendants, the local Dais should also be oriented in the techniques for safe and clean home delivery and in recognizing the common obstetric danger signs.

The messages in the IEEC materials emphasized the importance of prenatal care, good nutrition and iron supplementation during pregnancy, and the need for immediate transfer to the hospital if there were complications during pregnancy, delivery or the postpartum period. Including husbands in the IEEC improved women's use of prenatal care and increased the likelihood of reaching a hospital during an obstetric complication. In spite of the small sample size, there are indications that the husbands' IEEC helped to improve the general health and well-being of women during pregnancy, such as getting prenatal care, taking iron tablets and reducing household workload. Based upon these findings, we also recommend that behaviour change communication programs in maternal health should include a focus on husbands, particularly with regard to IEEC on obstetric care.

## List of abbreviations

CBI: Community-based intervention; DHS: Demographic and Health Surveys; EmONC: Emergency Obstetric and Neonatal Care; EPI: Expanded Programme of Immunization; IEEC: Information and education for empowerment and change; MCH: Maternal and child health; MDG: Millennium Development Goal; MMR: Maternal mortality ratio; MNH: Maternal and neonatal health; NGO: Non-governmental organization.

## Competing interests

The authors declare that they have no competing interests.

## Authors' contributions

This study was a component of an operations research project called Balochistan Safe Motherhood Initiative (BSMI). FM contributed to study design, field work, data analysis and writing of manuscript. SB contributed in study design, writing the grant proposal, data analysis and writing of manuscript.

## Author Information

*Farid Midhet *is currently Faculty Research Advisor at Qassim University College of Medicine, Saudi Arabia. He has worked before with Ministry of Health, Balochistan, Aga Khan University, The Asia Foundation and Population Council. He has MBBS from Pakistan, MPH from Columbia University (New York, USA) and DrPH from Johns Hopkins University (Baltimore, USA).

*Stan Becker *is a Professor at Johns Hopkins University. He has a PhD from Johns Hopkins University and an MA from the University of Chicago and has worked previously at the Centers for Disease Control, the International Centre for Diarrheal Disease Research, Bangladesh, the Institut National d'etudes Demographiques and the Virje Universiteit Brussel.

## Supplementary Material

Additional file 1 **Appendix**. Comparison of the characteristics of ever-married women of reproductive ages by study arm at Baseline Survey (1998)* Click here for file
