# Supplementary material for: Impact of community-based interventions on maternal and neonatal health indicators: Results from a community randomized trial in rural Balochistan, Pakistan
Source: Reprod Health. 2010 Nov 5;7:30. doi: 10.1186/1742-4755-7-30 (PMC2993657; doi:10.1186/1742-4755-7-30)
Supplement: Additional file 1 — Appendix. Comparison of the characteristics of ever-married women of reproductive ages by study arm at Baseline Survey (1998)* [file 1742-4755-7-30-S1.DOC]

# Additional files

**Appendix: Comparison of the characteristics of ever-married women of reproductive ages by study arm at Baseline Survey (1998)***

| Indicator | Control arm | Intervention arms | |
| --- | --- | --- | --- |
|  |  | Women’s IEEC only | Couples’ IEEC |
| Number of women in sample | 3,652 | 2,067 | 1,813 |
|  |  | Mean |  |
| Age at the time of survey (years) | 27.8 | 27.1 | 27.3 |
| Age at marriage | 14.3 | 14.1 | 14.3 |
| Number of children ever born | 4.6 | 4.5 | 4.5 |
| Currently alive children | 3.8 | 3.6 | 3.6 |
|  |  |  |  |
|  |  | % |  |
| Distribution of women by age-group  < 30 years  30-39 years  40-49 years | 60.7  27.7  11.6 | 63.8  27.7  9.4 | 62.7  26.8  9.4 |
| Distribution of women by parity (ns)  0-2 children  3-4 children  5 + children | 32.4  20.9  46.6 | 33.7  21.5  44.9 | 32.5  20.8  46.7 |
| Illiterate women in the sample | 94.6 | 94.9 | 95.2 |
| Illiterate husbands in the sample | 42.4 | 45.5 | 46.4 |
| Women working for pay or profit | 15.9 | 16.4 | 20.3 |
| Contraceptive prevalence rate | 5.8 | 5.0 | 6.7 |

*Differences of greater than 2.0 percentage points are statistically significant.
